# Glucose control after glucocorticoid administration in hospitalized patients – a retrospective analysis

**DOI:** 10.1186/s12902-021-00914-3

**Published:** 2022-01-05

**Authors:** Franzisca Merkofer, Tristan Struja, Neele Delfs, Carlos C. Spagnuolo, Jason F. Hafner, Kevin Kupferschmid, Ciril Baechli, Philipp Schuetz, Beat Mueller, Claudine A. Blum

**Affiliations:** 1 Department of General Internal Medicine, Medical University Clinic, Kantonsspital Aarau, Tellstrasse 25, Haus 7, 5001 Aarau, Switzerland; 2 Department of Endocrinology, Diabetology and Clinical Nutrition, Medical University Clinic, Kantonsspital Aarau, Tellstrasse 25, Haus 7, 5001 Aarau, Switzerland

**Keywords:** Diabetes, Glucocorticoids, Insulin, In-hospital hyperglycemia, Glucocorticoid-induced diabetes, Glucocorticoid-induced hyperglycemia

## Abstract

**Background:**

Glucocorticoid (GC)-induced hyperglycemia is a frequent adverse effect in hospitalized patients. Guidelines recommend insulin treatment to a target range of 6–10 mmol/L (108–180 mg/dl), but efficacies of particular regimes have not been well-studied.

**Methods:**

In this retrospective cohort study, hospitalized patients receiving GCs at the medical ward were analyzed by treatment (basal-bolus vs. bolus-only vs. pre-mixed insulin) and compared to a non-insulin-therapy reference group. Coefficients of glucose variation (CV), percentage of glucose readings in range (4–10 mmol/L (72–180 mg/dl)) and hypoglycemia (< 4 mmol/L (< 72 mg/dl)) were evaluated.

**Results:**

Of 2424 hospitalized patients receiving systemic GCs, 875 (36%) developed GC-induced hyperglycemia. 427 patients (17%) had a previous diagnosis of diabetes. Adjusted relative risk ratios (RRR) for the top tertile of CV (> 29%) were 1.47 (95% Cl 1.01–2.15) for bolus-only insulin, 4.77 (95% CI 2.67–8.51) for basal-bolus insulin, and 4.98 (95% CI 2.02–12.31) for premixed insulin, respectively.

Adjusted RRR for percentages of glucose readings in range were 0.98 (95% Cl 0.97–0.99) for basal-bolus insulin, 0.99 (95% Cl 0.98–1.00) for premixed insulin, and 1.01 (95% Cl 1.00–1.01) for bolus-only insulin, respectively. Adjusted RRR for hypoglycemia was 13.17 (95% Cl 4.35–39.90) for basal-bolus insulin, 8.92 (95% Cl 2.60–30.63) for premixed insulin, and 2.99 (95% Cl 1.01–8.87) for bolus-only insulin, respectively.

**Conclusions:**

Current guidelines recommend a basal-bolus regimen for treatment of GC-induced hyperglycemia, but we found similar outcomes with pre-mixed and bolus-only insulin regimens. As GC-induced hyperglycemia is a frequent issue in hospitalized patients, it might be reasonable to prospectively study the ideal regimen.

**Supplementary Information:**

The online version contains supplementary material available at 10.1186/s12902-021-00914-3.

## Background

Glucocorticoids (GCs) are used therapeutically to treat various diseases due to their immunosuppressive effect [1]. Despite their benefits, adverse effects are common. GC-induced hyperglycemia has an approximate incidence of 30% [2]. It is caused by several mechanisms including insulin resistance, increased gluconeogenesis and beta-cell dysfunction [3]. In-hospital hyperglycemia is generally associated with several adverse events such as higher in-hospital mortality, increased length of stay, and transfer to intensive care units [4]. Therefore, optimized diabetes therapy is crucial.

The current guidelines for the treatment of in-hospital hyperglycemia also cover the management of GC-induced hyperglycaemia. As such, the same target range and management is proposed as for all other causes of in-hospital hyperglycemia [5]. Over the last 10 years, the guidelines have changed from a rather tight control of blood glucose levels to modified regimens with at the lower ranges.

This change of paradigm is based on studies showing that a tight control, albeit leading to fewer infections, has no benefits in terms of mortality, myocardial infarction or stroke [5]. Adversely, stricter control entails an increased risk of hypoglycemia [5–7]. For persistent GC-induced hyperglycemia, basal-bolus insulin therapy is recommended [7, 8].

Hence, we retrospectively investigated different treatment strategies in hospitalized patients with GC-induced hyperglycemia. We were especially interested whether glucose control and hypoglycemia differed depending on the treatment strategy.

## Methods

### Setting and patients

This was a retrospective observational study conducted at the Medical University Clinic in Aarau, Switzerland. The retrospective analysis was done using electronic health record (EHR) data from hospitalized patients treated with GCs between January 2014 and April 2018. The Ethics Committee (EKNZ, Ethics Committee of Northwestern and Central Switzerland) approved the study (No. 2018–01271). The study adheres to the principles of the Declaration of Helsinki and to the STROBE statement. Written informed consent from each patient for the use of EHR data for research was granted by January 1st 2018, as required by local legislation. The Ethics Committee approved use of retrospective data for this analysis in the time frame prior to this date.

Inclusion criteria were age of at least 18 years, a hospital stay of at least 3 days, stay at a medical ward, administration of at least 10 mg prednisolone (or its equivalent), and for hospitalizations after January 1st 2018 general consent for the use of EHR. The sole exclusion criterion was a recurrent admission within 30 days of the index hospitalization. GC dose, indication for GC administration, glucose measurements, insulin dosage, previous diagnosis and treatment of diabetes, and hospital events were evaluated. Interviews conducted 30 days after discharge for quality control reasons were available for evaluation.

### Standard of care at the studied hospital

At our center, the standard of care after GC administration included blood glucose measurements at 10:00 a.m. and at 2:30 p.m. in order to screen for GC-induced hyperglycemia. This includes a standardized electronic insulin protocol with a low threshold of prescribing bolus insulin, in order to cover meals with an assumed additional need of insulin during GC administration, as published elsewhere [9] and daily screening for hyper- and hypoglycemia by endocrinology service is done on weekdays. We also used a low threshold of prescribing bolus insulin, in order to cover meals with an assumed additional need of insulin during GC administration [9].

If GC-induced hyperglycemia occurred, the subsequent treatment choice was made individually, based on hypoglycemia risk, comorbidities, and patient resources to administer the treatment regimen after discharge.

As a local standard, dosage of bolus insulin was adjusted to carbohydrate intake. Basal-bolus insulin usually consisted of insulin detemir twice per day and a rapid acting insulin analogue (insulin lispro or insulin aspart). Typically, the amount of basal and bolus insulin was balanced evenly. In case of pre-mixed insulin treatment, a 50:50 mixture of insulin lispro protamine suspension and insulin lispro injection (HUMALOG® Mix 50/50™ or similar) was used. Two thirds of the total daily premixed insulin dose was generally administred before breakfast, while the remainder, one-third, was injected before lunch.

### Outcomes and endpoints

The objective of this study was to evaluate the differences in glucose control between different treatment strategies for GC-induced hyperglycemia. Primary endpoints were glycemic variability, measured by coefficient of variation (CV), percentage of glucose values in the range of 4–10 mmol/L (72–180 mg/dl), and hypoglycemia below 4.0 mmol/L (72 mg/dl). Secondary endpoints were cumulative GC-dose, duration of GC administration, indication for GC administration, mean glucose values and mean insulin administration per patient according to body weight and day.

### Hypothesis

We hypothesized that due to individualized treatment, the differences in blood glucose control between the different treatment regimens would be small, but that hypoglycemia rate and CV would be higher in more intensly treated groups.

### Statistical analysis

Discrete variables are expressed as counts (percentages) and continuous variables either as means and standard deviations (SD) or as medians and interquartile ranges (IQR).

Continuous variables were analyzed using ANOVA (Analysis of Variance) or Wilcoxon rank-sum test. Categorical and binary variables were analyzed using Chi-square test.

The following three treatment groups were compared to a no insulin treatment reference group: bolus-only insulin, basal-bolus insulin, and premixed insulin.

We evaluated associations between treatment strategy, blood glucose control, and outcomes using a multinomial (polytomous) logistic regression model taking the no insulin treatment group as reference. First, we calculated an unadjusted model, one for each of the three outcomes glycemic variability (glucose CV), percentage of glucose values in range, and hypoglycemia. The CV for glycemic variability was evenly distributed into tertiles and compared to the first tertile as reference.

In a second model, we adjusted each of the outcomes for length of hospital stay (LOS), glucose levels at hospital entry, age, Charlson Comorbidity Index [10], cumulative GC-dose per kilogram of body weight per GC administration day, GC-induced hyperglycemia, and preexisting diabetes.

Significance level was set to an alpha of 5%. All statistical tests were two-sided. We performed no adjustment for multiple testing. The data was analyzed using Stata v15.1 (StataCorp, Texas, USA).

### Definitions

GC-induced hyperglycemia was defined as morning fasting blood glucose of > 7.0 mmol/L (126 mg/dl) or a random blood glucose of > 11.0 mmol/L (198 mg/dl) after the start of GCs. Normoglycemia was defined as fasting blood glucose of 7.0 mmol/L (126 mg/dl) or lower, and random or postprandial blood glucose readings of 11.0 mmol/L (198 mg/dl) or lower. GC-induced diabetes was defined by fulfilling the above criteria without a preexisting diabetes. Day 1 was defined as the first day of GC administration. Glycemic variability was calculated as CV = SD/mean*100. The percentage of glucose values in range was defined as the number of finger stick glucose measurements between 4.0–10.0 mmol/L (72–180 mg/dl). Any blood glucose < 4 mmol/L (< 72 mg/dl) was defined as hypoglycemia. Severe hypoglycaemia was defined as blood glucose < 2.5 mmol/L (< 45 mg/dl). The GC dose was assessed as mg prednisolone equivalent per kg body weight per GC administration day.

Reasons for GC administration were grouped as following: autoimmune/inflammation (neuroinflammatory disease, rheumatologic disease including gout and vasculitis, allergology/dermatology, chronic inflammatory bowel diseases, kidney transplantation, and glomerulonephritis), hemato-oncology (chemotherapy, antiemetics, treatment of side effects or local tumor compression), infection/pneumology (chronic obstructive pulmonary disease (COPD), respiratory infections, sepsis) and endocrinology (mainly adrenal insufficiency).

## Results

### Baseline characteristics

**Fig. 1:**
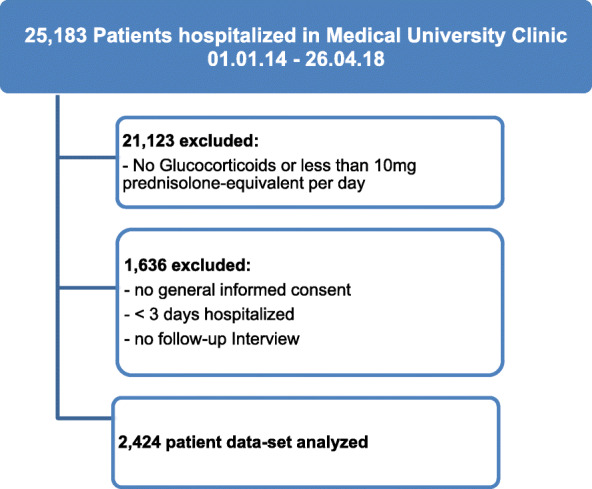
STROBE study flow diagram

Of the 25,183 hospitalized patients during the studied time range, 16% of patients (n = 4060) received GCs of at least 10 mg prednisolone equivalent. 1636 patients had to be excluded from the study either due to a LOS of less than 3 days (n = 986), no given general consent (n = 109) or no follow-up interview (n = 541). Data from 2424 patients were available for analysis (see Fig. 1 for study flow chart).

1123 (46%) were men, median age was 69 years (IQR 59–78), and 511 patients (21%) had a pre-existing diabetes (see Table 1 for baseline characteristics).

**Table 1:** Baseline Characteristics*

	Total	Basal & bolus insulin	Pre-mixed insulin only	Bolus only	No insulin therapy	p-value
	N = 2424	N = 277	N = 122	N = 652	N = 1373	
Male sex	1123 (46.3%)	115 (41.5%)	48 (39.3%)	306 (46.9%)	654 (47.6%)	0.11
Age	69.4 (59.0–77.7)	70.0 (61.7–77.0)	74.6 (65.7–81.8)	70.4 (59.2–77.5)	68.2 (56.8–77.7)	< 0.001
BMI [kg/m2]	25.2 (22.1–29.0)	27.4 (24.3–32.6)	26.2 (24.0–30.2)	25.7 (22.4–29.9)	24.5 (21.4–27.9)	< 0.001
Cumulative GC-dose, mg†	200.0 (100.0–375.0)	200.0 (100.0–411.2)	250.0 (145.0–525.0)	245.0 (140.0–522.5)	175.0 (100.0–300.0)	< 0.001
GC dose dose, mg per day†	50.0 (27.5–76.6)	50.0 (26.9–70.2)	50.0 (25.0–81.2)	50.0 (35.0–88.9)	50.0 (25.0–75.0)	< 0.001
Duration of GC administration, days	5.0 (3.0–7.0)	5.0 (3.0–7.0)	6.0 (4.0–8.0)	5.0 (3.0–7.0)	4.0 (3.0–6.0)	< 0.001
Length of stay, days	8.0 (5.0–14.0)	10.0 (6.0–16.0)	10.0 (6.0–15.0)	8.0 (5.0–13.0)	8.0 (5.0–13.0)	< 0.001
Indication for GC administration						
- Autoimmune/Inflammation	816 (33.7%)	99 (35.7%)	51 (41.8%)	220 (33.7%)	446 (32.5%)	< 0.001
- Oncology	667 (27.5%)	60 (21.7%)	23 (18.9%)	149 (22.9%)	435 (31.7%)	
- Infection/Pneumology	825 (34.0%)	101 (36.5%)	42 (34.4%)	253 (38.8%)	429 (31.2%)	
- Endocrinology	116 (4.8%)	17 (6.1%)	6 (4.9%)	30 (4.6%)	63 (4.6%)	
Charlson Comorbidity Index	2.0 (1.0–4.0)	2.0 (1.0–3.0)	2.0 (1.0–3.0)	2.0 (1.0–3.0)	2.0 (1.0–4.0)	0.37
Pre-existing diabetes	511 (21.1%)	216 (78.0%)	84 (68.9%)	157 (24.1%)	54 (3.9%)	< 0.001

*The “no insulin treatment” group served as reference group

† given as prednisone equivalent in mg

GC: Glucocorticoid

Data are presented as median (IQR) for continuous measures, and n (%) for categorical measures

Median cumulative GC dose was 175 mg (IQR 100–300) in patients with no insulin treatment, 245 mg (IQR 140–523) in patients with bolus-only insulin, 200 mg (IQR 100–411) in patients with basal-bolus insulin, and 250 mg (IQR 145–525) in patients with premixed insulin. Duration of GC treatment was 4.0 (3.0–6.0) days for patients with no insulin treatment, 5.0 (3.0–7.0) for bolus-only insulin, 5.0 (3.0–7.0) days for basal-bolus insulin, and 6.0 (4.0–8.0) days in patients with premixed insulin, respectively.

Preexisting diabetes was present in 54 (4%) patients without insulin, 157 (24%) patients with bolus-only insulin, 216 (78%) patients with basal-bolus insulin, and 84 (69%) patients with premixed insulin.

Overall, 875 (36%) patients developed GC-induced hyperglycemia. 488 (18%) patients in total developed a new-onset GC-induced diabetes, of which 144 (10%) did not receive an insulin treatment, 229 (35%) patients had bolus-only insulin, 41 (14%) patients had basal-bolus insulin, and 34 (35%) premixed insulin (see also Table 2).

**Table 2:** Glucose levels and insulin dosing

	Total	Basal & bolus insulin	Pre-mixed insulin only	Bolus only	No insulin therapy*	p-value
	N = 2424	N = 277	N = 98	N = 652	N = 1397	
No. of Glucose readings per patient	5.00 (0.00–12.00)	22.00 (13.00–31.00)	19.50 (12.00–28.00)	9.00 (6.00–14.00)	0.00 (0.00–4.00)	< 0.001
Glucose coefficient of variation	0.25 (±0.10)	0.33 (±0.09)	0.33 (±0.11)	0.24 (±0.09)	0.21 (±0.08)	< 0.001
% of glucose readings in range^†^	66.67 (42.86–83.33)	34.78 (21.43–55.56)	42.86 (30.00–65.62)	71.43 (50.00–86.67)	75.00 (53.85–100.00)	< 0.001
Mean fasting glucose day 1–7	6.14 (5.40–7.30)	7.91 (6.35–9.57)	7.37 (6.44–9.15)	5.90 (5.33–6.80)	5.70 (5.20–6.47)	< 0.001
Mean preprandial glucose day 1–7^§^	8.46 (7.29–10.70)	11.55 (9.35–13.12)	10.98 (8.91–12.24)	8.20 (7.05–9.79)	7.60 (6.81–8.37)	< 0.001
Mean postprandial glucose day 1–7^||^	8.50 (7.20–10.38)	11.04 (9.15–13.47)	11.06 (9.10–13.30)	8.05 (7.05–9.44)	7.80 (6.70–8.90)	< 0.001
Hypoglycemia < 4.0 mmol/L	89 (3.67%)	53 (19.13%)	13 (13.27%)	18 (2.76%)	5 (0.36%)	< 0.001
Hypoglycemia < 2.5 mmol/L	8 (0.33%)	6 (2.17%)	1 (1.02%)	0 (0.00%)	1 (0.07%)	< 0.001
Mean insulin/patient/kg weight/day	0.07 (0.03–0.18)	0.21 (0.13–0.31)	0.21 (0.15–0.30)	0.04 (0.02–0.07)	0.12 (0.06–0.32)	< 0.001
Normoglycemia	1549 (63.90%)	27 (9.75%)	3 (3.06%)	295 (45.25%)	1224 (87.62%)	< 0.001
GC-induced hyperglycemia	875 (36.10%)	250 (90.25%)	95 (96.94%)	357 (54.75%)	173 (12.38%)	< 0.001
- New-onset GC-induced diabetes	448 (18.48%)	41 (14.80%)	34 (34.69%)	229 (35.12%)	144 (10.31%)	
- Preexisting diabetes	427 (17.62%)	209 (75.45%)	61 (62.24%)	128 (19.63%)	29 (2.08%)	

Data are presented as mean (±SD) or median (IQR) for continuous measures, and n (%) for categorical measures

* The “no insulin therapy” group served as a reference group

† Target range: 4–10 mmol/L (72–180 mg/dl)

‡ at 8:00 a.m.

§ at 12:00 p.m. and 6:00 p.m.

|| at 2:00 a.m., 10:00 a.m., 2:00 p.m., 8:00 p.m., 10:00 p.m.

CV: Coefficient of variation

GC: Glucocorticoid

Data are shown as n (%) or mean (SD) if not mentioned otherwise

### Glucose levels, insulin dosing, and hyperglycemia

Mean fasting blood glucose value was 5.7 mmol/L (IQR 5.2–6.47) with no insulin treatment, 5.9 mmol/L (5.33–6.80) with bolus-only insulin, 7.91 mmol/L (IQR 6.35–9.57) with basal-bolus insulin, and 7.37 mmol/L (IQR 6.44–9.15) with premixed insulin. The CV of glucose was 21% (±0.08%) for the no insulin treatment group, 24% (±0.09%) for bolus-only insulin, 33% (±0.09%) for basal-bolus insulin, and 33% (±0.11%) for premixed insulin. The percentage of glucose values in range in the no insulin treatment group was 75% (IQR 54–100%), 71% (IQR 50–87%) with bolus-only insulin, 35% (IQR 21–56%) with basal-bolus insulin, and 43% (IQR 30–66%) with premixed insulin.

The incidence of hypoglycemia < 4.0 mmol/L was 5 patients (0.4%) in the no insulin treatment group, 18 patients (2.8%) with bolus-only insulin, 53 patients (19.1%) with basal-bolus insulin, and 13 patients (13.3%) with premixed insulin (see also Table 2).

### Glycemic variability

The relative risk ratio (RRR) for a CV between 21 and 29% (middle tertile) in the unadjusted multinomial logistic regression was 1.53 (95% CI 1.17–2.00) with bolus-only insulin, 3.19 (95% Cl 1.96–5.20) with basal-bolus insulin, and 3.78 (95% Cl 1.71–8.34) with premixed insulin. For CV over 29%, the RRR was 2.37 (95% Cl 1.70–3.28) for bolus-only insulin, 20.62 (95% Cl 12.81–33.20) for basal-bolus insulin, and 22.54 (95% Cl 10.71–47.47) for premixed insulin.

**Fig. 2:**
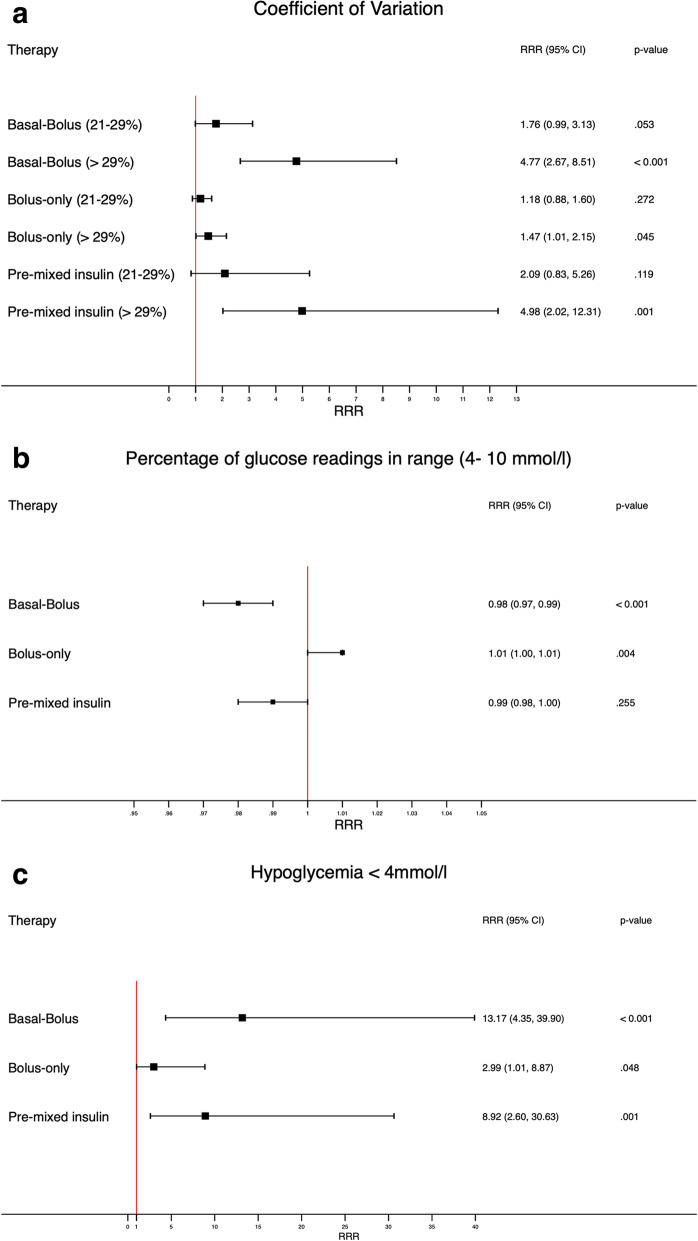
**A** Coefficient of glucose variation according to treatment group. **B** Percentage of glucose readings in range according to treatment group. **C** Risk of hypoglycemia according to treatment group

In the adjusted model, the RRR for a CV of glucose between 21 and 29% was 1.18% (95% Cl 0.88–1.60) for bolus-only insulin, 1.76% (95% Cl 0.99–3.13) for basal-bolus insulin, and 2.09% (95% Cl 0.83–5.26) for premixed insulin. For a CV of glucose > 29%, the RRR was 1.47 (95% Cl 1.01–2.15) with bolus-only insulin, 4.77 (95% Cl 2.67–8.51) with basal-bolus insulin, and 4.98 (95% Cl 2.02–12.31) with premixed insulin (see also Fig. 2A and supplemental Table 1). The CV of glucose was similar in patients with no insulin and bolus-only insulin. But in the basal-bolus group and the premixed insulin group, patients had similar but much higher CVs of glucose (see supplemental Fig. 1).

### Percentage of glucose readings in range

In the unadjusted multinominal logistic regression model, the RRR for a glucose value in range was 1.00 (95% Cl 0.99–1.00) with bolus-only insulin, 0.96 (95% Cl 0.95–0.96) with basal-bolus insulin, and 0.97 (95% Cl 0.96–0.97) with premixed insulin.

In the adjusted model, the RRR for a glucose value in range was 1.01 (95% Cl 1.00–1.01) with bolus-only insulin, 0.98 (95% Cl 0.97–0.99) with basal-bolus insulin, and 0.99 (95% Cl 0.98–1.00) with premixed insulin. The RRRs for percentage of glucose values in range in both GC-induced diabetes and preexisting diabetes were significantly higher with all three insulin treatments. (for detailed values, see Fig. 2B and supplemental Table 1).

### Rate of hypoglycemia

In the unadjusted multinominal logistic regression model, the RRR for hypoglycaemia was 7.90 (95% Cl 2.92–21.38) with bolus-only insulin, 65.87 (95% Cl 26.05–166.57) with basal-bolus insulin, and 42.83 (95% Cl 14.83–112.21) with premixed insulin.

In the adjusted model, the RRR was 2.99 (95% Cl 1.01–8.87) with bolus-only insulin, 13.17 (95% Cl 4.35–39.90) with basal-bolus insulin, and 8.92 (95% Cl 2.60–30.63) with premixed insulin. The RRRs for GC induced diabetes and preexisting diabetes were significantly higher with all three insulin treatments. (see Fig. 2C and supplemental Table 1 for detailed values). Hypoglycemia was more frequent with higher CV of glucose (see supplemental Fig. 2).

## Discussion

In this retrospective observational study, we found that patients with higher glycemic variability tended to have lower glucose values in target range and had a higher relative risk of hypoglycemic events. These two characteristics were more prominent in patients who received basal-bolus or premixed insulin than in patients who received bolus insulin with meals or for correction only. When comparing basal-bolus insulin with premixed insulin, glucose variability was similar, but the premixed insulin group had an 8% higher percentage of glucose values in the target range, alongside a trend towards a lower risk of hypoglycemia (19.1% vs. 13.3%). Although mean insulin per patient was 0.12 E/kg body weight, patients in the bolus-only group needed just 0.05 E/kg of body weight, whereas patients in the basal-bolus group and the premixed insulin group needed 0.23 E/kg body weight and 0.24 E/kg body weight, respectively. As a limitation, we cannot exclude that this allegedly favorable effect of premixed insulin was based on a preselection of suitable patients. Guidelines for hospital hyperglycemia recommend basal-bolus insulin for hyperglycemia but state that for short duration of GCs prandial insulin or intermediate-acting insulin may be sufficient, while more severe hyperglycemia may need to be treated with more complex insulin regimens [7, 8]. Several randomized controlled trials (RCTs) [11–13] and a recent systematic review [14] advocate premixed or intermediate-acting insulin. The rationale for premixed or intermediate-acting insulin is based on the pharmacologic effect of prednisone on glucose metabolism, the most frequently used GC. Prednisone is generally expected to have a 16-h effect, with a peak after 4–6 h, with low morning glucose, high postprandial values due to glucose intolerance, and a decreasing effect of insulin resistance after 16 h, i.e. in the evening, with a predisposition to evening or nocturnal hypoglycemia [14]. Lakhani et al. showed that with an additional correction accounting for dose, type of GC, and patient body weight, better glucose control is possible [15].

Two RCTs investigated the additional use of oral antidiabetics in hospitalized patients with GC-induced hyperglycemia [16, 17]. Long-term data of metformin for the prevention of metabolic complications of GCs have shown some benefits [18, 19], but feasibility in hospitalized patients has only been investigated in a small trial [17]. For mild GC-induced hyperglycemia, metformin or other oral antidiabetics are promising, although further studies are warranted to show not only safety, but also a beneficial short-term effect. Our own data were too heterogenous to assess the effect of oral antidiabetics on glucose control.

### Limitations and strengths

Our work has several limitations. These result primarily from the fact that the data were from a single center with similar providers and standard operating procedures, thus these standard operating procedures without randomized treatment allocation may have led to confounding by indication. There is also a risk of residual bias from unmeasured factors. Therefore, our results should be interpreted with caution and should serve as the basis for designing a thorough randomized controlled trial. In addition, the relatively small number of patients diagnosed with GC-induced diabetes prohibits a separate analysis of this subgroup. In addition, the fact that blood glucose levels were measured only twice per day is certainly a limitation. It is possible that hypoglycemia could almost have been noticed earlier and could have been treated or prevented earlier. The relatively small number of patients also resulted in a wide confidence interval. Therefore, the results must be interpreted with caution when comparing the groups.

One strength of this study lies in the relatively large number of complete data sets for analysis and that it bases on real-life data, despite its retrospective character. Another strength is that it is one of the first comprehensive reviews investigating associations between treatment strategy, blood glucose control and outcomes.

## Conclusions

We found two phenotypes of GC-induced hyperglycemia: a milder type which may be treated with no or low doses of insulin, and a severe type which requires basal-bolus or premixed insulin. In the severe type, basal-bolus and premixed insulin were similarly efficient for achieving treatment targets. Current guidelines recommend a basal-bolus regimen for treatment of GC-induced hyperglycemia. As GC-induced hyperglycemia is a frequent issue in hospitalized patients, it might be reasonable to prospectively study the ideal regimen. We are aware that the results of our retrospective work can be used to influence practice. However, they can guide planning of a sufficiently powered randomized controlled trial which in turn provides reassuring evidence to influence guidelines for patients GC-induced hyperglycemia.

## Supplementary Information

**Additional file 1: Supplemental Table 1.** Results of the unadjusted and adjusted multinomial logistic regression. **Supplemental Fig. 1.** Glucose variability according to treatment strategy. **Supplemental Fig. 2.** Glucose variability in patients with and without hypoglycemia.

## Data Availability

The datasets used and/or analysed during the current study are available from the corresponding author on reasonable request.

## Abbreviations

COPD: Chronic obstructive pulmonary disease
CV: Coefficient of variation, calculated as SD/mean*100%
GC: Glucocorticoid
EHR: Electronic health record
LOS: Length of hospital stay
RCT: Randomized controlled trial
RRR: relative risk ratios
SD: Standard deviation
