# Supplementary material for: Glucose control after glucocorticoid administration in hospitalized patients – a retrospective analysis
Source: BMC Endocr Disord. 2022 Jan 5;22:8. doi: 10.1186/s12902-021-00914-3 (PMC8734262; doi:10.1186/s12902-021-00914-3)
Supplement: Supplementary file 1 — Additional file 1: Supplemental Table 1. Results of the unadjusted and adjusted multinomial logistic regression. Supplemental Fig. 1. Glucose variability according to treatment strategy. Supplemental Fig. 2. Glucose variability in patients with and without hypoglycemia. [file 12902_2021_914_MOESM1_ESM.docx]

Additional Material

Supplemental Table 1. Results of the unadjusted and adjusted multinomial logistic regression

Supplemental Figure 1. Glucose variability according to treatment strategy

Supplemental Figure 2. Glucose variability in patients with and without hypoglycemia

Supplemental Table 1. Results of the unadjusted and adjusted multinomial logistic regression

| Unadjusted model | Bolus only insulin | p-value | Basal-bolus insulin | p-value | Pre-mixed Insulin | p-value |
| --- | --- | --- | --- | --- | --- | --- |
| *Outcome 1:* |  |  |  |  |  |  |
| CV of glucose |  |  |  |  |  |  |
| 0-0.206 |  |  | Ref. |  |  |  |
| 0.206-0.288 | 1.53 (1.17 - 2.00) | 0.002 | 3.19 (1.96 - 5.20) | 0.000 | 3.78 (1.71 - 8.34) | 0.001 |
| >0.288 | 2.37 (1.70 - 3.28) | 0.000 | 20.62 (12.81 - 33.20) | 0.000 | 22.54 (10.71 - 47.47) | 0.000 |
| *Outcome 2:* |  |  |  |  |  |  |
| % of glucose readings in range 4-10 mmol/L (72-180 mg/dl) | 1.00 (0.99 - 1.00) | 0.161 | 0.96 (0.95 - 0.96) | 0.000 | 0.97 (0.96 - 0.97) | 0.000 |
| *Outcome 3:* |  |  |  |  |  |  |
| Hypoglycemia < 4.0mmol/L (72 mg/dl) | 7.90 (2.92 - 21. 38) | 0.000 | 65.87 (26.05 - 166.57) | 0.000 | 42.58 (14.83 - 112.21) | 0.000 |
|  |  |  |  |  |  |  |
| Adjusted model |  |  |  |  |  |  |
| *Outcome 1:* |  |  |  |  |  |  |
| CV of glucose |  |  |  |  |  |  |
| 0-0.206 |  |  | Ref. |  |  |  |
| 0.206-0.288 | 1.18 (0.88 - 1.60) | 0.272 | 1.76 (0.99 - 3.13) | 0.053 | 2.09 (0.83 - 5.26) | 0.199 |
| >0.288 | 1.47 (1.01 - 2.15) | 0.045 | 4.77 (2.67 - 8.51) | 0.000 | 4.98 (2.02 - 12.31) | 0.001 |
| *Covariates:* |  |  |  |  |  |  |
| Length of stay | 0.99 (0.97 - 1.00) | 0.068 | 1.00 (0.99 - 1.02) | 0.617 | 1.01 (1.00 - 1.03) | 0.118 |
| Glucose on admission | 1.03 (0.97 - 1.10) | 0.353 | 1.20 (1.12 - 1.29) | 0.000 | 1.21 (1.12 - 1.31) | 0.000 |
| Age | 1.00 (0.99 - 1.01) | 0.378 | 1.00 (0.98 - 1.01) | 0.804 | 1.04 (1.02 - 1.07) | 0.001 |
| Charlson Comorbidity Index | 1.03 (0.98 - 1.08) | 0.201 | 1.09 (1.02 - 1.17) | 0.014 | 1.01 (0.91 - 1.12) | 0.907 |
| GC dose | 1.00 (0.95 - 1.06) | 0.875 | 0.99 (0.91 - 1.08) | 0.827 | 1.05 (0.95 - 1.15) | 0.369 |
| GC-induced diabetes | 1.62 (1.20 - 2.19) | 0.002 | 3.77 (2.24 - 6.35) | 0.000 | 8.63 (3.14 - 23.75) | 0.000 |
| Pre-existing diabetes | 2.13 (1.45 - 3.12) | 0.000 | 10.86 (6.76 - 17.45) | 0.000 | 5.86 (3.20 - 10.73) | 0.000 |
| *Outcome 2:* |  |  |  |  |  |  |
| % of glucose readings in range 4-10 mmol/L (72-180 mg/dl) | 1.01 (1.00 - 1.01) | 0.004 | 0.98 (0.97 - 0.99) | 0.000 | 0.99 (0.98 - 1.00) | 0.255 |
| *Covariates:* |  |  |  |  |  |  |
| Length of stay | 0.99 (0.97 - 1.00) | 0.105 | 1.01 (0.99 - 1.03) | 0.203 | 1.02 (1.00 - 1.04) | 0.078 |
| Glucose on admission | 1.05 (0.98 - 1.12) | 0.105 | 1.18 (1.10 - 1.27) | 0.000 | 1.21 (1.12 - 1.31) | 0.000 |
| Age | 1.00 (0.99 - 1.01) | 0.596 | 1.00 (0.98 - 1.01) | 0.612 | 1.04 (1.02 - 1.06) | 0.001 |
| Charlson Comorbidity Index | 1.03 (0.98 - 1.08) | 0.204 | 1.09 (1.02 - 1.17) | 0.016 | 1.00 (0.90 - 1.11) | 0.977 |
| GC dose | 1.00 (0.95 - 1.06) | 0.908 | 0.97 (0.89 - 1.06) | 0.488 | 1.03 (0.94 - 1.14) | 0.489 |
| GC-induced diabetes | 2.23 (1.67 - 2.97) | 0.000 | 5.51 (3.34 - 9.11) | 0.000 | 14.12 (5.27 - 37.82) | 0.000 |
| Pre-existing diabetes | 2.37 (1.62 - 3.46) | 0.000 | 9.19 (5.74 - 14.72) | 0.000 | 5.94 (3.21 - 10.98) | 0.000 |
| *Outcome 3:* |  |  |  |  |  |  |
| Hypoglycemia < 4.0mmol/L (72 mg/dl) | 2.99 (1.01 - 8.87) | 0.048 | 13.17 (4.35 - 39.90) | 0.000 | 8.92 (2.60 - 30.63) | 0.001 |
| *Covariates:* |  |  |  |  |  |  |
| Length of stay | 0.99 (0.98 - 1.00) | 0.094 | 1.00 (0.98 - 1.02) | 0.994 | 1.01 (1.00 - 1.03) | 0.133 |
| Glucose on admission | 1.05 (0.99 - 1.11) | 0.120 | 1.24 (1.16 - 1.33) | 0.000 | 1.26 (1.17 - 1.35) | 0.000 |
| Age | 1.00 (0.99 - 1.01) | 0.852 | 0.99 (0.98 - 1.01) | 0.302 | 1.04 (1.01 - 1.06) | 0.002 |
| Charlson Comorbidity Index | 1.00 (0.96 - 1.04) | 0.895 | 1.05 (0.98 - 1.12) | 0.142 | 0.97 (0.88 - 1.07) | 0.565 |
| GC dose | 1.03 (0.97 - 1.08) | 0.338 | 1.00 (0.98 - 1.10) | 0.846 | 1.07 (0.97 - 1.17) | 0.186 |
| GC induced diabetes | 4.69 (3.61 - 6.09) | 0.000 | 14.08 (8.64 - 22.95) | 0.000 | 34.38 (13.99 - 90.94) | 0.000 |
| Pre-existing diabetes | 2.88 (2.01 - 4.11) | 0.000 | 13.93 (8.79 - 22.09) | 0.000 | 7.64 (4.21 - 13.87) | 0.000 |

The “no insulin therapy” group served as a reference group. Results are given as relative risk ratios with corresponding 95% confidence intervals.

CV: Coefficient of variation

GC: Glucocorticoid


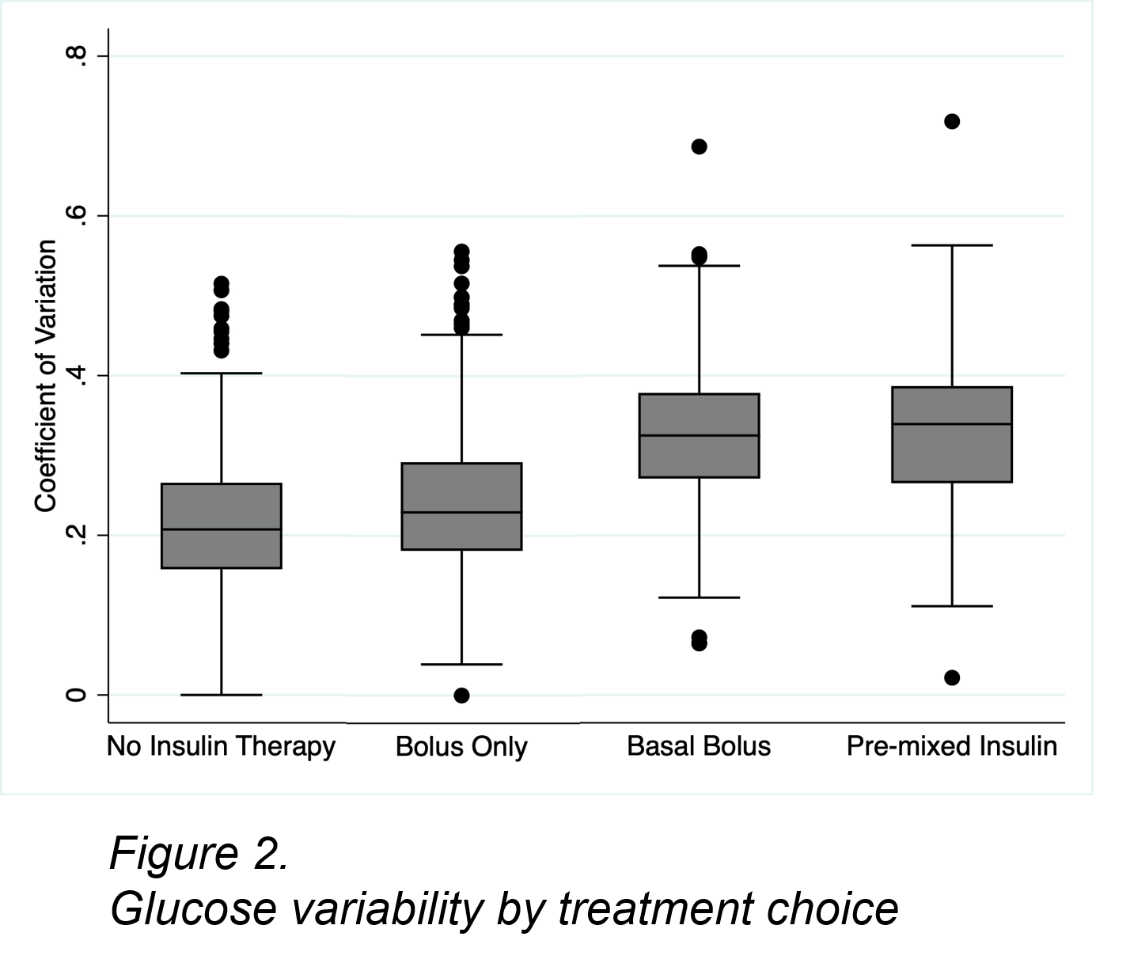


Supplemental figure 1. Glucose variability according to treatment strategy


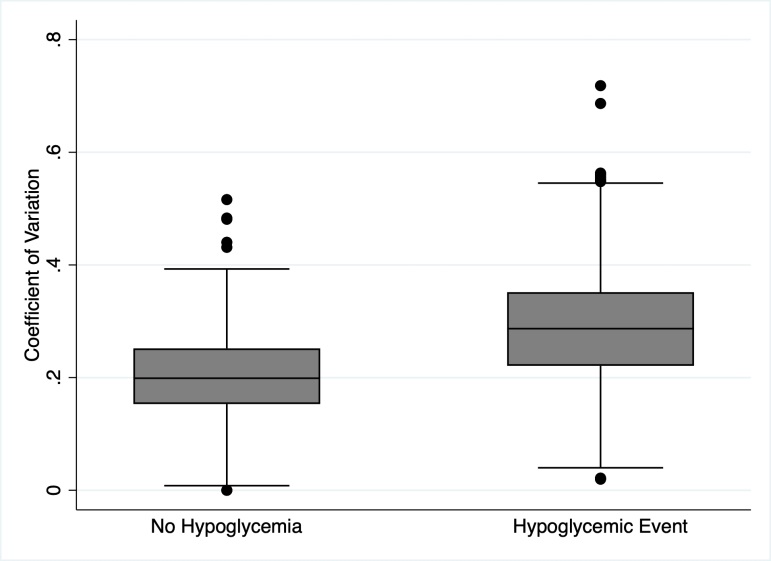


Supplemental figure 2. Glucose variability in patients with and without hypoglycemia
